# Petroleum-Tolerant Rhizospheric Bacteria: Isolation, Characterization and Bioremediation Potential

**DOI:** 10.1038/s41598-020-59029-9

**Published:** 2020-02-06

**Authors:** Jéssica Aparecida Viesser, Maura Harumi Sugai-Guerios, Lucca Centa Malucelli, Marcia Regina Pincerati, Susan Grace Karp, Leila Teresinha Maranho

**Affiliations:** 1 PhD Program in Industrial Biotechnology, Universidade Positivo (UP), Av. Pedro Prof. Viriato Parigot de Souza, 5300, CEP 81280-330, Curitiba, PR Brazil; 2 PhD Program in Environmental Management, Universidade Positivo (UP), Av. Pedro Prof. Viriato Parigot de Souza, 5300, CEP 81280-330, Curitiba, PR Brazil; 3 PhD Program in Bioprocess Engineering and Biotechnology, Universidade Federal do Paraná, Caixa Postal 19011 – ACF Centro Politécnico, CEP 81531-980, Curitiba, PR Brazil

**Keywords:** Environmental biotechnology, Environmental microbiology

## Abstract

Petroleum is an important energy source. Due to its intensive exploration, accidents resulting in oil spills on soil are frequent, which creates consequences to ecosystems and human health. Rhizodegradation is an efficient technique that promotes the decontamination of polluted environments through the selection and use of rhizosphere microorganisms from phytoremediation plants. The aim of this study was to isolate, identify and characterize bacteria capable of degrading petroleum from the rhizosphere of *Panicum aquaticum* Poir., a plant that grows in petroleum contaminated soils. Three bacteria were isolated and characterized at the morphological (Gram staining), molecular (16S rRNA gene sequence analysis) and biochemical level. These bacteria were identified as new strains of *Bacillus thurigiensis*, *Bacillus pumilus* and *Rhodococcus hoagii*, which have been reported as potential bioremediators in the literature. All three bacteria were able to use petroleum hydrocarbons as the sole carbon source during i*n vitro* degradation assays. Gas chromatography analysis of these assays indicated reductions of petroleum hydrocarbons between 23% and 96% within 48 h. Among the isolated bacteria, *Rhodococcus hoagii* presented the highest efficiency of petroleum consumption, reaching 87% of degradation after only 24 h of cultivation, which corresponds to a higher and faster degradation than previously reported, confirming the potential use of *Rhodococcus hoagii* for petroleum biodegradation.

## Introduction

Petroleum has a complex composition formed predominantly by aliphatic and aromatic hydrocarbons, and lower concentrations of asphaltenes, resins and metals. In turn, petroleum composition varies according to the geographical localization and physical, chemical and biological conditions of the environment where it is formed^1^. Many compounds present in petroleum are toxic, mutagenic or carcinogenic^2^. The effects of naphthalene in humans, for example, include skin irritation, red blood cells breakdown and nephrotoxicity^2,3^.

Petroleum spills into the environment result from the high volume of petroleum used as raw material for energy and chemicals production as well as the accidents during operating processes, transportation, refining, storage and consumption^4,5^. In contact with the environment, petroleum undergoes changes in its original characteristics, due to the interaction between physical and biological factors^6,7^. The persistence of petroleum hydrocarbons in the environment is a result of their slow biodegradation and can compromise quality of water resources and the soil and may be accumulated in food, such as vegetables, muscles and fish^2,8^.

Environment-friendly approaches have been proposed to remediate petroleum-contaminated environments. According to Pandey *et al*.^9^ and Paul *et al*.^10^, bioremediation is widely used in environmental decontamination due to its relatively lower costs and higher efficiency when compared to chemical and physical remediation techniques. Bioremediation is based on the metabolization of pollutants through their complete removal, immobilization or transformation into less toxic products, which consequently mitigate their toxic effects in the environment^11,12^. In phytoremediation, the interaction between plants and rhizospheric microorganisms is used to degrade, remove or stabilize contaminants in soil and water^5,13^.

The tolerance of plants in soils contaminated with organic pollutants, such as petroleum, occurs primarily through the association of microorganisms to the roots. When stimulated (phytostimulation), these become responsible for the biodegradation of hydrocarbons, in a process called rhizodegradation^13,14^. The petroleum hydrocarbons are consumed by the rhizospheric organisms as carbon source, converting them into carbon dioxide (CO_2_), water and biomass^15^.

Among the various plants used in phytoremediation, the species of the family Poaceae show potential for use in decontamination of petroleum-contaminated soils. This is because they can reduce erosion and increase organic matter content in the soil^16^, while presenting rapid growth and branching roots that provide a larger surface area for the colonization of rhizospheric microorganisms^13,17^. Among the Poaceae, genus *Panicum* has been reported to reduce petroleum hydrocarbons in contaminated soil, for example, *Panicum virgatum* L. (switchgrass) was able to remove 37% of pyrene added to the soil, converting it into CO_2_ (32%) and plant biomass (5%)^18,19^. *Panicum* is a cosmopolitan genus with wide distribution around the world and mainly present in cultivated fields, low-density forests and grass fields^20^.

The aim of this work was to isolate, identify and evaluate the petroleum degradation potential of three rhizospheric bacteria from *Panicum aquaticum* cultivated in soil contaminated with petroleum. *Panicum aquaticum* Poir, popularly known as turtle grass, is a Brazilian native species mainly found in very humid locations^21^.

## Material and Methods

### Enrichment of rhizospheric bacteria capable of degrading petroleum

In order to facilitate the isolation of rhizospheric bacteria that are capable of degrading petroleum in contaminated soils, an enrichment method was used. This method consisted on collecting uncontaminated soil; artificially contaminating this soil with petroleum; cultivating *P. aquaticum* in the contaminated soil; and isolating active bacteria from the resulting rhizospheric soil. It is expected that the bacteria present in the original soil sample that are unable to degrade petroleum do not survive the artificial contamination of the soil. Meanwhile, petroleum degrading bacteria present in the original soil sample are expected to survive and divide while *P. aquaticum* is cultivated in the contaminated soil. Therefore, active bacteria in the resulting rhizospheric soil should be able to degrade petroleum in soil environments.

For the enrichment method, uncontaminated soil was collected at the margins of Iraí reservoir at Pinhais (state of Paraná, Brazil), coordinates - 25,3890012 and -49,0959464. This is a natural site for *P. aquaticum*, thus the soil contained microorganisms able to adhere to this plant. Ten samples of soil were collected at a depth of 20 cm and homogenized (pH 5.40, 0.00 cmolc Al^3+^.dm^−3^; 4.60 cmolc H^+^ + Al^3+^.dm^−3^; 4.60 cmolc Ca^2+^.dm^−3^; 3.10 cmolc Mg^2+^.dm^−3^; 1.11 cmolc K^+^.dm^−3^; cation exchange capacity 18.51 cmolc.dm^−3^; 262.50 mg Phosphorus.dm^−3^; 52.20 g Carbon.dm^−3^; base saturation 75.00%; exchangeable Al 0.00%; 425.00 g clay.kg^−1^; 3.50 g total Nitrogen.kg^−1^).

In the laboratory, soil samples were contaminated with 100 g of petroleum kg^−1^ of soil. *Panicum aquaticum* were cultivated in this contaminated soil in a greenhouse for 90 days, when plants were 25 cm high and their roots were 30 cm long. In order to distinguish rhizosphere soil from bulk soil, only soil adhering to plant roots were used in the isolation step.

### Isolation and morphological characterization of bacteria

For the isolation, 2.5 g of soil adhering to plant roots were transferred to 250 mL of sterile 0.1% (w/v) peptone solution. This mixture was incubated in orbital shaker TE-420 (Tecnal, Brazil) at 36 °C and 145 rpm for 24 h. Serial dilutions were performed, and the dilutions 10^−6^, 10^−7^ and 10^−8^ were inoculated in Nutrient Agar (NA). Three colonies distinct morphologies were isolated in NA plates^22^. Isolated colonies were numbered 1 to 3 and kept in NA plates. Semi-permanent slides were prepared by Gram staining for morphological characterization of the colonies^23^.

### Molecular characterization

Genomic DNA was extracted through a phenol/chloroform method adapted by Thomaz-Soccol *et al*.^24^, which is the stablished protocol in our laboratory. Samples were incubated with lysis buffer (Tris-EDTA pH 8.0, SDS and Proteinase K) for 2 h at 56 °C, followed by proteinase denaturation at 95 °C for 10 min. RNAse was added and the mixture was incubated at 37 °C for 2 h. Saturated phenol was added and samples were centrifugated for 5 min at 20 °C and 10.000 g. Supernatant was collected, a second phenol extraction from the pellet was performed and the supernatant from both centrifugation steps were combined. Phenol/chloroform solution was added to the supernatants and samples were centrifugated at 10.000 g for 5 min at 20 °C. This procedure was repeated twice and supernatants were collected. DNA from the supernatants was precipitated with the addition of 3 M sodium acetate and 96% ethanol at 4 °C, followed by incubation for 18 h at −20 °C and centrifugation at 10.000 g for 30 min at 4 °C. The remaining pellet was washed twice with 70% ethanol, dried for 10 min at 37 °C and DNA was resuspended in 100 μL of water.

Purity and DNA concentration in the resulting solution were determined by absorbance at 260 nm in a NanoDrop 2000c spectrophotometer (Thermo Scientific, USA). The 16S rRNA gene was amplified by polymerase chain reaction (PCR) using primers 27 F (5′ AGTTTGATCCTGGCTCAG 3′) and 1492 R (5′ ACGGCTACCTTGTTACGACTT 3′)^25,26^. PCR amplification was carried out in a final volume of 20 µL, which was composed of 50 mM KCl, 20 mM Tris-HCl (pH 8.4), 1.5 mM MgCl_2_, 4.0 mM dNTP mix, 2.5 mM of each primer, 1.5 U *Platinum Taq* DNA Polymerase (Invitrogen) and 50 ng of genomic DNA. DNA amplification was performed in a thermal cycler (MasterCycler, Germany), at the following conditions: 95 °C for 5 min (denaturation), followed by 35 cycles of 94 °C for 1 min, 58 °C for 1 min (annealing), 72 °C for 1 min (extension), and a final extension at 72 °C for 5 min. The amplification products were verified by electrophoresis in 1.5% (w/v) agarose gel, stained with ethidium bromide and visualized on UV transilluminator.

Subsequently, the PCR products were purified and sequenced on an automated sequencer (3500 Genetic Analyzer, USA) using the same predesigned primers. The obtained 16S rDNA sequences were analyzed for similarity using the Basic Local Alignment Search Tool (BLAST) database. The sequences were also analyzed using the Ribosomal Database Project (RDP) (https://rdp.cme.msu.edu/) for confirmation. A phylogenetic tree for each strain was generated with Blast (http://www.ncbi.nlm.nih.gov/Blast/) with default parameters, in which phylogenetic analyses were performed by fast minimum evolution algorithm. Resulting gene sequences were deposited at the GenBank.

### Biochemical characterization of *Bacillus* species

*Bacillus thuringiensis*, *B. cereus* and other *Bacillus* species present similar 16S rDNA sequences and differentiation between these species requires phenotypic analysis through a staining method using 0.5% hot basic fuchsin^27^. This technique is based on the differentiation of colonies through the detection of endotoxin crystals, which are produced by *Bacillus thuringiensis*, but not by other species from the *B. cereus* group.

### Petroleum degradation assays

The petroleum degradation potential was analyzed based on the methodology proposed by Mishra *et al*.^28^. using 25 mL of mineral medium (0.5 g L^−1^ K_2_HPO_4_; 0.5 g L^−1^ (NH_4_)_2_SO_4_; 0.5 g L^−1^ MgSO_4_.7H_2_O; 0.01 g L^−1^ FeCl_3_; 0.001 g L^−1^ MnCl_2_; 0.0001 g L^−1^ ZnSO_4_; 0.01 g L^−1^ CaCl_2_) adapted from Déziel *et al*.^29^, supplemented with 1% (v/v) of petroleum as the only carbon source, as proposed by Omotayo *et al*.^30^. For the inoculated treatment (IT), which corresponds to the bacterial biodegradation, mineral medium supplemented with petroleum was inoculated with each bacterial strain at a final concentration of 10^8^ cells mL^−1^. A control treatment (CT) was conducted without inoculation, thus corresponding to natural attenuation or evaporation of hydrocarbons present in the medium. Flasks were incubated in orbital shaker at 36 °C and 145 rpm for different time intervals (0 h, 24 h, 48 h, 72 h and 96 h), in triplicate.

At each time interval, the following parameters were analyzed for both treatments (CT and IT): pH using pHmeter TCP01 (Onda Científica, Campinas, Brazil), dissolved oxygen (DO) using oximeter Handylab OX12-Set (SI Analytics, Mainz, Germany), electrical conductivity (EC) with Handylab LF11 (SI Analytics, Mainz, Germany) and biomass concentration. Biomass concentration was determined with a gravimetric method adapted from Makkar and Cameotra^31^. In brief, 5 mL of each sample (in duplicate) were centrifuged at 12000 *g* for 20 min and the resulting pellet was oven dried at 105 °C until constant weight.

### Gas chromatography

The efficiencies of petroleum degradation for each bacterial strain were evaluated by extraction and quantification of total petroleum hydrocarbons (TPH) during the petroleum degradation assays, according to the method proposed by Schwab *et al*.^32^. This method was selected because the authors demonstrated that this is a simple and efficient method for the extraction of petroleum hydrocarbons from soil, comparing it to other methods with different sample types, extraction conditions and extraction solvents. For each strain, samples were taken after 24 h and 48 h of incubation from the CT and the IT treatments. For each sample, 1 g anhydrous Na_2_SO_4_ was added to absorb water and 5 mL of dichloromethane (analytical standard) was added for the extraction of petroleum compounds in a magnetic stirrer for 1 h. Then, solution was centrifuged at 1120 g for 15 min. Supernatant was transferred to amber glass bottles and stored at 4 °C until evaluation by gas chromatography (GC).

The samples were analyzed in GC using the liquid/gas chromatograph Shimadzu GC-2010 (Japan) with capillary column DB-5 (0.25 µm diameter, 30 m length and 0.25 µm width). The injector and detector temperatures were 250 °C and 280 °C, respectively. Hydrogen was used as the carrier gas (1.0 mL min^−1^). The column temperature program was as follows: the temperature was set to 70 °C for 4 min and raised to 190 °C (20 °C min^−1^), then to 250 °C (10 °C min^−1^) and finally to 280 °C (30 °C min^−1^). A temperature hold was set for another 10 min. Sample’s injection was performed in triplicate using 0.5 µL of sample. Twelve peaks were selected and the percentage of area reduction was quantified using CT samples as a point of reference for the remaining compounds (100%) in the untreated system, since the incubation with agitation may result in a reduction of hydrocarbons concentration due to evaporation.

### Statistical analysis

Data were analyzed for normality with the Shapiro-Wilk test. Since they presented normal distribution, they were compared using the parametric t-Student test. The nonparametric results were compared using the Kruskal-Wallis (H) test. Parameters pH, T, EC, DO, B and OD were compared between different incubation times and CT and IT treatments for each strain. The Spearman’s rank correlation (r) was applied to examine relationships between the parameters. The reference values that qualified the correlations were adopted in accordance with Callegari-Jacques^33^: 0 < r < 0.3 (low), 0.3 ≤ r < 0.6 (moderate) and r ≥ 0.6 (strong). All statistical analyzes were performed with the Statistica software, version 10.0, at a confidence level of 95%.

## Results

### Morphological and molecular characterization of isolated bacteria

**Figure 1:**
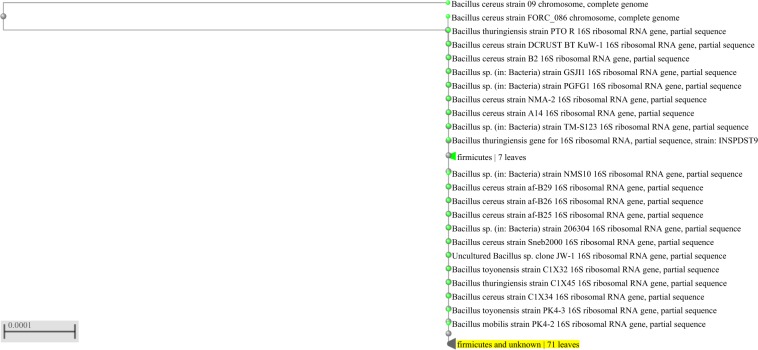
Phylogenetic tree showing close homologs of strain 1- *Bacillus thuringiensis*.

**Figure 2:**
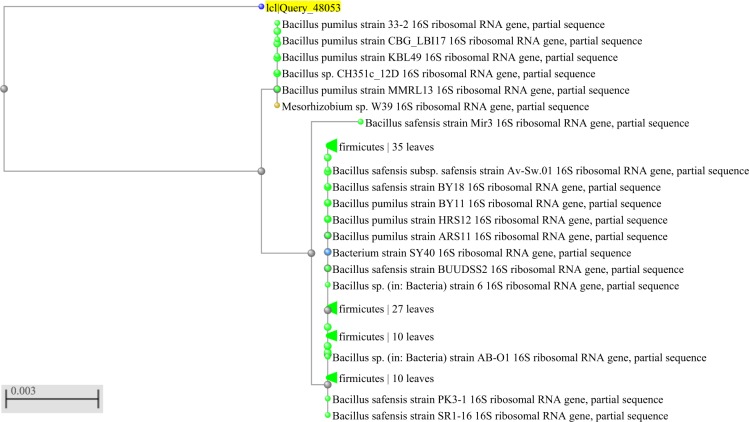
Phylogenetic tree showing close homologs of strain 2 - *Bacillus pumilus*.

**Figure 3:**
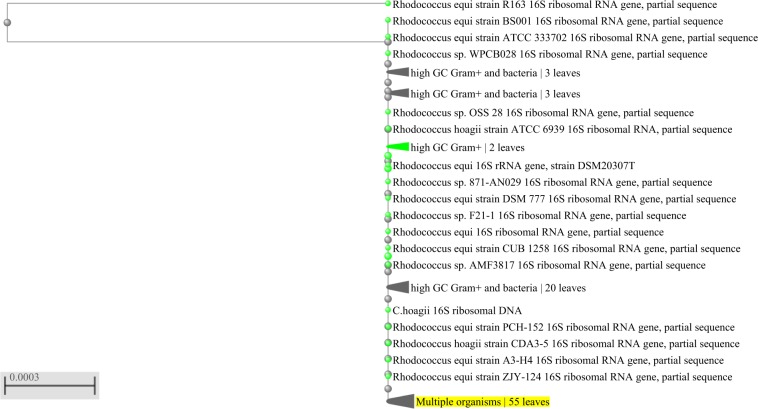
Phylogenetic tree showing close homologs of strain 3 - *Rhodococcus hoagie*.

Three rhizospheric bacteria were isolated from *P. aquaticum* and classified as Gram-positive. In relation to their morphology, two were identified as bacilli and one as coccus (Table 1). The nucleotide sequences of the 16S rDNA (Figs. 1, 2 and 3) enabled the identification at the species level of strain 2, which was identified as *Bacillus pumilus* (Genbank accession number: MN625237) with 98.29% of identity (Fig. 2), and strain 3, which was identified as *Rhodococcus hoagii* (previously known as *Rhodococcus equi*, Genbank accession number: MN625254) with 99.49% of identity (Fig. 3). Based on nucleotide sequences of the 16S rDNA, strain 1 was classified as a member of *Bacillus cereus* group (Genbank accession number: MN625145) with 99.88% of identity (Fig. 1). Strain 1 was identified as *B. thuringiensis* through the hot basic fuchsin staining, based on the presence of bipyramidal pink crystalline inclusion bodies outside the lysed cells, which occurs only in *B. thuringiensis*.

**Table 1:** Morphological characterization of bacteria isolated from rhizosphere of *Panicum aquaticum* Poir.

Molecular ID	Colony color in NA	Gram	Morphology
*Bacillus thurigiensis*	White	Positive	Rod
*Bacillus pumilus*	Beige	Positive	Rod
*Rhodococcus hoagii*	Light Pink	Positive	Coccus

### Petroleum biodegradation assay

Each strain was incubated in mineral medium supplemented with 1% (v/v) of petroleum as the only carbon source. The values of pH (Table 2) showed statistical significance between CT and IT. For IT, the pH remained between 5.86 and 7.06, and the highest value of biomass was around pH 7.0. The most significant pH variation was the pH reduction from 6.74 at 48 h to 5.86 at 96 h for *Rhodococcus hoagii*. Chen *et al*.^34^. reported that the growth of microorganisms is favored in a pH range between 6.0 and 8.0. Balachandran *et al*.^6^ found that the highest degradation rates of naphthalene (99.14%), diesel oil (98.25%) and phenanthrene (17.50%) by *Streptomyces* sp. took place at pH 7.0.

**Table 2:** pH, dissolved oxygen (DO), electrical conductivity (EC) and biomass concentration (B) during petroleum degradation assays with Bacillus thuringiensis, Bacillus pumilus and Rhodococcus hoagii at different cultivation periods.

	Cultivation time (h)	pH		DO (mg L^−1^)		EC (mS cm^−1^)		B (g L^−1^)	
		CT	IT	CT	IT	CT	IT	CT	IT
*Bacillus thuringiensis*	0	6.58 ± 0.20 ^aA^	6.74 ± 0.14^bA^	1.49 ± 0.11^bA^	1.24 ± 0.13 ^aA^	1678.67 ± 57.83^bcA^	5.65 ± 0.08^bB^	0.00 ± 0.00^aB^	1.43 ± 0.03^bA^
	24	6.71 ± 0.13^aB^	7.06 ± 0.01 ^aA^	1.26 ± 0.03^cA^	0.72 ± 0.06^bB^	1737.00 ± 89.94^bA^	5.76 ± 0.17^bB^	0.00 ± 0.00^aB^	6.20 ± 0.42 ^aA^
	48	6.85 ± 0.19 ^aA^	7.04 ± 0.05 ^aA^	1.80 ± 0.04 ^aA^	0.79 ± 0.06^bB^	1843.33 ± 37.90^abA^	6.02 ± 0.05^aB^	0.00 ± 0.00^aB^	7.58 ± 0.54 ^aA^
	72	6.90 ± 0.09 ^aA^	6.87 ± 0.06 ^aA^	1.74 ± 0.09 ^aA^	0.61 ± 0.16^bB^	1864.67 ± 52.79^abA^	6.12 ± 0.13^aB^	0.00 ± 0.00^aB^	4.61 ± 1.19 ^aA^
	96	6.79 ± 0.10 ^aA^	6.89 ± 0.12 ^aA^	1.42 ± 0.04^bcA^	0.67 ± 0.06^bB^	1940.67 ± 43.82 ^aA^	6.82 ± 0.38^aB^	0.00 ± 0.00^aB^	4.03 ± 0.57 ^aA^
*Bacillus pumilus*	0	6.68 ± 0.21^bA^	6.73 ± 0.09 ^aA^	1.37 ± 0.03 ^aA^	0.60 ± 0.26^aB^	1618.00 ± 209.36 ^aA^	5.48 ± 0.15^bB^	0.01 ± 0.01^aB^	1.03 ± 0.74^bA^
	24	6.52 ± 0.20^bA^	6.59 ± 0.15 ^aA^	0.98 ± 0.05^bA^	0.49 ± 0.29^aB^	1651.00 ± 104.92 ^aA^	5.48 ± 0.17^bB^	0.00 ± 0.00^aB^	2.82 ± 1.62 ^aA^
	48	6.56 ± 0.16^bA^	6.82 ± 0.25 ^aA^	0.84 ± 0.14^bA^	0.44 ± 0.10^aB^	1788.33 ± 150.32 ^aA^	6.19 ± 0.10^aB^	0.00 ± 0.00^aB^	3.29 ± 0.62 ^aA^
	72	7.43 ± 0.23 ^aA^	6.65 ± 0.25^aB^	0.95 ± 0.07^bA^	0.82 ±± 0.15 ^aA^	1567.67 ± 91.98 ^aA^	6.21 ± 0.50^aB^	0.00 ± 0.00^aB^	2.98 ± 0.24 ^aA^
	96	6.47 ± 0.22^bA^	6.60 ± 0.13 ^aA^	0.96 ± 0.08^bA^	0.44 ± 0.05^aB^	1598.67 ± 243.67 ^aA^	6.82 ± 0.09^aB^	0.00 ± 0.00^aB^	3.83 ± 0.18 ^aA^
*Rhodococcus hoagii*	0	6.92 ± 0.26 ^aA^	6.46 ± 0.38 ^aA^	2.52 ± 0.23 ^aA^	2.19 ± 0.15^bA^	1796.67 ± 63.96 ^aA^	5.90 ± 0.54^aB^	0.02 ± 0.01^aB^	1.70 ± 0.64 ^aA^
	24	6.59 ± 0.15 ^aA^	6.62 ± 0.38 ^aA^	2.01 ± 0.20 ^aA^	2.02 ± 0.53^bA^	1797.67 ± 60.75 ^aA^	5.77 ± 0.57^aB^	0.05 ± 0.15^aB^	2.50 ± 0.33 ^aA^
	48	6.59 ± 0.57 ^aA^	6.74 ± 0.31 ^aA^	2.10 ± 0.15 ^aA^	1.94 ± 0.20^bA^	1759.33 ± 10.97 ^aA^	6.11 ± 0.08^aB^	0.00 ± 0.00^aB^	3.10 ± 0.88 ^aA^
	72	6.60 ± 0.14 ^aA^	6.25 ± 0.19 ^aA^	2.48 ± 0.20^aB^	3.96 ± 0.21 ^aA^	1860.33 ± 42.00 ^aA^	7.37 ± 2.07^aB^	0.00 ± 0.00^aB^	2.93 ± 0.88 ^aA^
	96	6.41 ± 0.02 ^aA^	5.86 ± 0.15^bB^	2.40 ± 0.28^aB^	3.17 ± 0.41 ^aA^	1755.67 ± 50.46 ^aA^	6.13 ± 0.12^aB^	0.08 ± 0.02^aB^	2.53 ± 0.54 ^aA^

Averages followed by the same lowercase letters in the same column do not differ statistically by Tukey’s test (p < 0.05). Averages followed by the same uppercase letters in the same row do not differ statistically by Tukey’s test (P < 0.05). Non-parametric data were analyzed through Kruskal-Wallis test (p < 0.05). CT is control treatment, which corresponds to the medium without bacteria, and IT is inoculated treatment.

**Figure 4:**
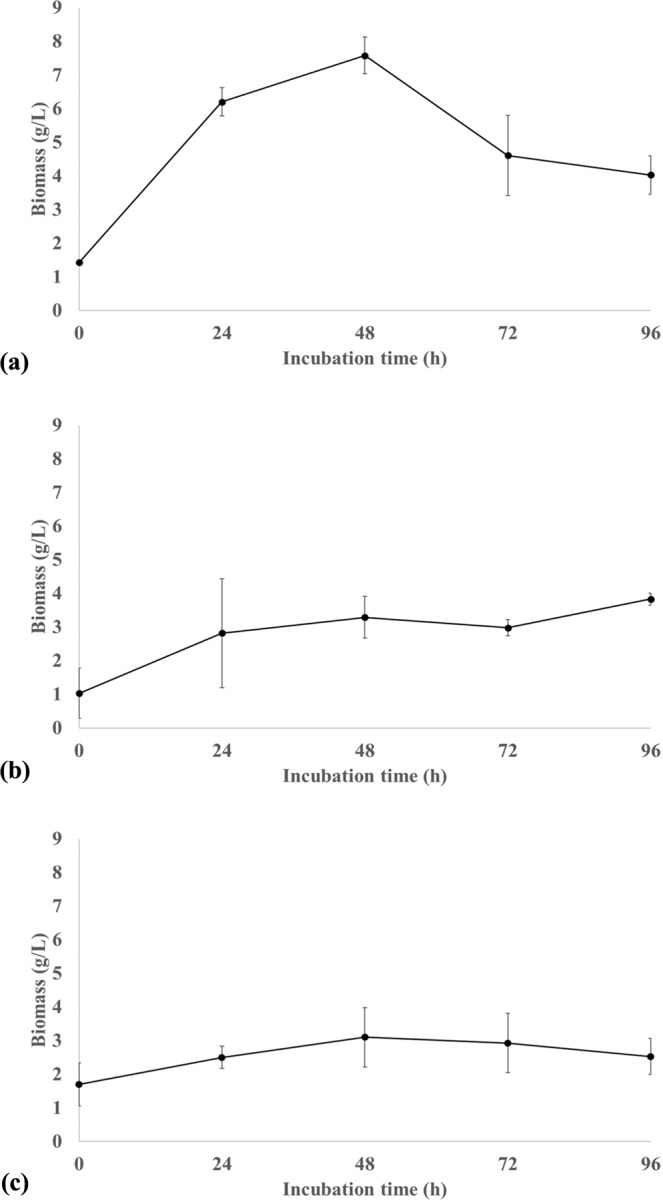
Biomass concentration during cultivation in mineral medium with 1% (v/v) of petroleum with (**a**) *Bacillus thuringiensis*, (**b**) *Bacillus pumilus* and (**c**) *Rhodococcus hoagii*.

Among the bacteria tested, *B. thuringiensis* reached the highest biomass concentration, 7.58 g L^−1^, at 48 h of incubation (Fig. 4). After 48 h, biomass concentration of *B. thuringiensis* decreased to 4.03 g L^−1^ at 96 h of cultivation indicating cellular death. *B. pumilus* reached the highest biomass concentration of 3.83 g L^−1^ at 96 h, however, biomass concentration between 24 h and 96 h were not statistically different (Table 2). *R. hoagii* reached the highest biomass concentration of 3.10 g L^−1^ at 48 h, however, biomass concentrations throughout the experiment were not statistically different, indicating low biomass production (Table 2). For 48 h of incubation, the lowest biomass productivity was obtained with *R. hoagii*, 0.0348 g L^−1^ h^−1^, followed by *B. pumilus* with 0,0471 g L^−1^ h^−1^, which is approximately one third of productivity reached with *B. thuringiensis*, 0.128 g L^−1^ h^−1^. Low biomass production obtained with *B. pumilus* and *R. hoagii* indicates that the petroleum hydrocarbons were degraded by the bacteria and used to generate metabolites instead of being incorporated into the biomass.

In relation to the dissolved oxygen (DO) content, O_2_ consumption was detected only in the experiment with *B. thuringiensis*, which is the strain with the highest growth rate. Oxygen concentration is a limiting factor for aerobic bacteria growth and for the decomposition of petroleum hydrocarbons by enzymatic cleavage (oxygenases) of their aromatic rings^11,35^, whose products are used in energy generation pathways^11,36^. Deng *et al*.^37^, when investigating the potential of petroleum hydrocarbons degradation by *Achromobacter* sp, observed that the highest degradation rate occurred during reactor media agitation, thus allowing sufficient oxygen transfer and maintaining the optimum DO levels for biodegradation.

The EC was used to monitor the consumption of nutrients present in the medium (Table 2). The CT showed higher EC when compared to IT, demonstrating the intense consumption of nutrients by microorganisms. In *B. thuringiensis* and *B. pumilus* degradation assays, it was observed an increase in ions content during the 96 h while for *R. hoagii* there were oscillations in ion levels. Atekwana *et al*.^38^ reported that the increase in electrical conductivity can be related to petroleum degradation, because the bacteria consume nutrients, forming dissociated ions and thereby increasing the electrical conductivity. Also, EC increase may be related to the secretion of metabolites by the microorganism or cell lysis.

**Figure 5:**
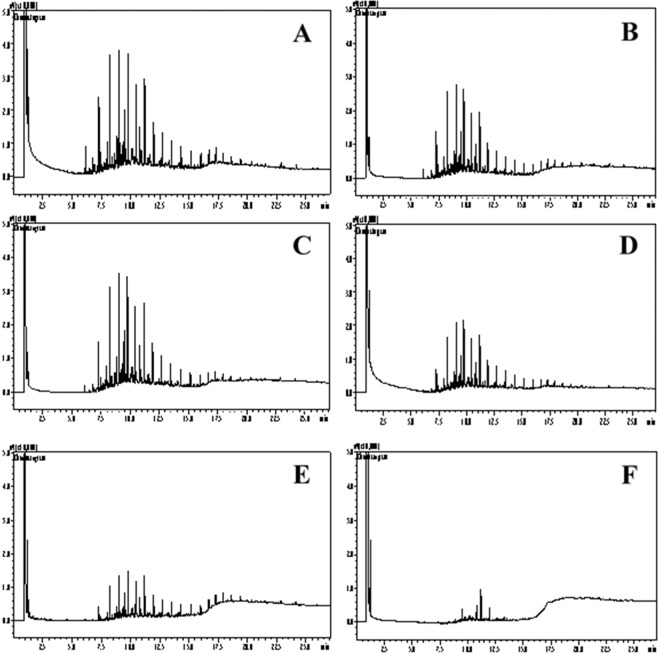
Chromatograms for the petroleum degradation assay with (**a**,**b**) *Bacillus thuringiensis*, (**c**,**d**) *Bacillus pumilus* and (**e**,**f**) *Rhodococcus hoagii* at (**a**,**c**,**e**) 24 h and at (**b**,**d**,**f**) 48 h of incubation.

Most of biomass growth occurred between 0 h and 48 h, therefore samples for 24 h and 48 h of cultivation were used to determine degradation of petroleum hydrocarbons through GC analysis of TPH. Chromatograms analysis indicate a reduction in petroleum compounds by the selected bacteria (Fig. 5).

**Figure 6:**
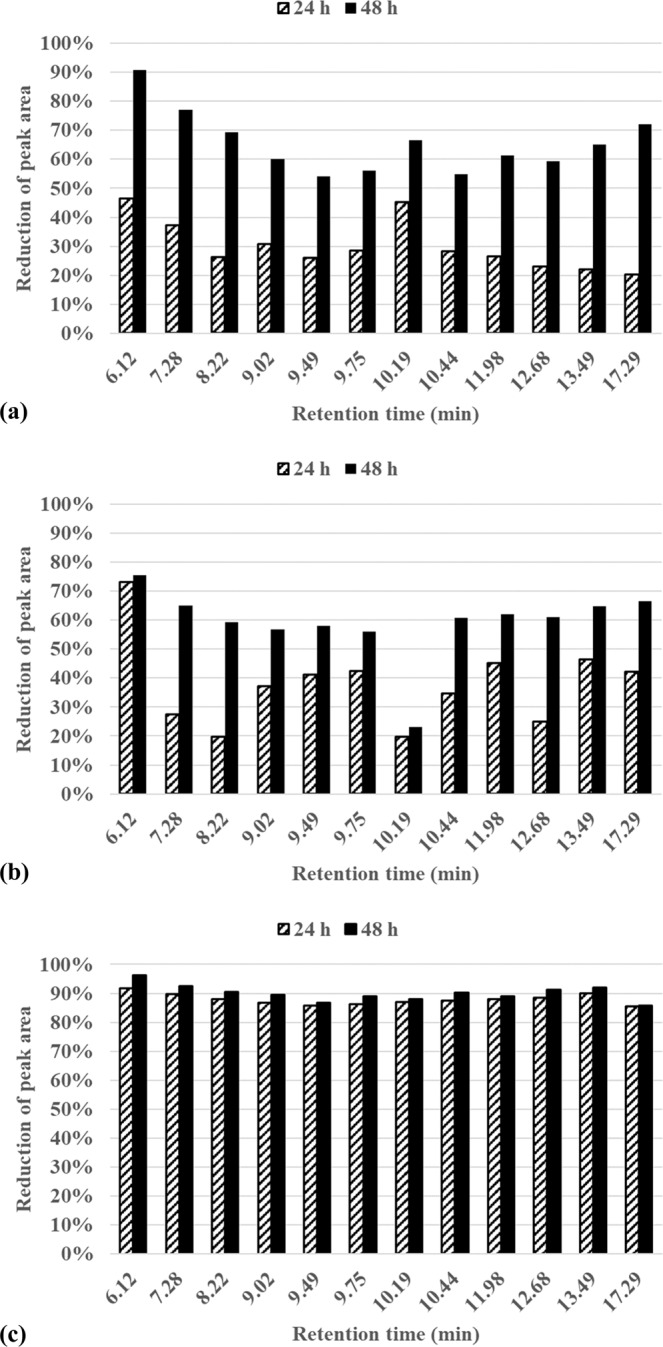
Reduction of peak areas during cultivation in mineral medium with 1% (v/v) of petroleum with (**a**) *Bacillus thuringiensis*, (**b**) *Bacillus pumilus* and (**c**) *Rhodococcus hoagii* at 24 h and 48 h of incubation.

Efficiency in biodegradation of petroleum compounds was analyzed based on reduction of peak areas of the compounds compared to the control treatment (Fig. 6; Supplementary Table S1), as suggested by Zhang *et al*.^39^. *R. hoagii* demonstrated the highest efficiency for the degradation of TPH regardless of the chain length (represented by retention time), with its efficiency for 24 h of incubation being higher than the efficiencies for 48 h of incubation with *B. thuringiensis* and *B. pumilus*. The polar fraction of petroleum is highly resistant to microbial degradation^4^, but light (aliphatic) hydrocarbon chains, such as n-alkanes, are more biodegradable than aromatic compounds^40^. Nevertheless, petroleum degradation efficiency for *R hoaggi* at 24 h was above 85% for all analyzed peaks, indicating that this strain is capable of degrading a variety of petroleum hydrocarbons. From 24 h to 48 h of incubation, the increase in degradation efficiency with *R hoaggi* was small, with the efficiency reaching between 85.83% and 96.15%, therefore, 24 h of incubation with *R hoaggi* is sufficient for the degradation of most of the TPH. Analyzing degradation efficiency (Fig. 6c) combined with biomass production (Fig. 4c), TPH degradation by *R. hoagii* appears to be independent of biomass growth. *R. hoagii* presents a great potential for bioremediation purposes since it can provide fast and effective degradation of TPH with low biomass production.

For *B. thurigiensis*, biodegradation of TPH improved significantly between 24 h and 48 h of incubation (Fig. 6a). Among the peaks analyzed, degradation efficiency varied from 20.32% to 46.62% at 24 h and from 54.17% to 90.77% at 48 h of incubation. Although all compounds had a degradation efficiency of more than 50% at 48 h of incubation, only the peak with the lowest retention time had a degradation efficiency higher than 80%. Since biomass concentration decreases after 48 h, indicating cellular death, it is unlikely that longer incubation time reaches the same degradation efficiency achieved with *R. hoagii*. Efficiencies obtained for *B. thurigiensis* at 24 h of incubation are the only data set in the present work in which there is a correlation between degradation efficiency and retention time, with the highest efficiencies corresponding to the lowest retention times. On the other hand, this correlation is not detected in the data for 48 h of incubation with *B. thurigiensis*.

*B. pumilus* was the least efficient strain because, even after 48 h of incubation, degradation efficiencies were between 23.05% and 75.56% (Fig. 6b). In addition, between 24 h and 48 h of incubation, degradation efficiency was almost the same for peak of retention time of 6.12 min and of 10.19 min, even though there is a significant increase for other peaks (e.g. 7.28 min), indicating that this strain is not able to fully degrade the TPH in the assay conditions.

## Discussion

In the present work, *Bacillus thuringiensis*, *Bacillus pumilus* and *Rhodococcus hoagii* were isolated from rhizosphere of *P. aquaticum* cultivated in petroleum-contaminated soil and were able to degrade TPH in laboratory assays. These genera of bacteria have already been described as capable of surviving in contaminated soils. For example, Wolinska *et al*.^41^ isolated and identified bacteria present 42 days after soil was contaminated with either 95-octane petrol or diesel. In the soil contaminated with petrol, *Bacillus*, *Microbacterium* and *Rhodococcus* species were found, whereas *Arthrobacter*, *Paenibacillus*, *Pseudomonas* and *Rhodococcus* species were found in the soil contaminated with diesel. However, the only species that was isolated in both works is *Bacillus pumilus*.

The efficiency of petroleum and other hydrocarbons degradation by the genus *Bacillus* is widely reported, especially due to the ability of these microorganisms to produce biosurfactants^4^. Biosurfactant producing bacteria can be found in hydrocarbons-contaminated sites^42,43^. *B. pumilus* has been described as a producer of lipopeptide surfactin, responsible for reducing the surface and interfacial tension^44^. Among the strains isolated in the present work, *Bacillus pumilus* presented the lowest TPH degradation efficiency, however, it may be used in a consortia of bacteria due to its surfactant production or it may be used in other remediation processes, for example, heavy metals removal^45^.

The microorganism *B. thuringiensis* is a widely used species in the production of biopesticides, but few studies have demonstrated its potential for degradation of pollutants such as petroleum. Maiti *et al*.^46^ investigated *B. thuringiensis* isolated from a petroleum-contaminated site and reported that the maximum degradation of the polycyclic aromatic hydrocarbons (PAHs) fluoranthene and pyrene occurred in the presence of Tween 80, a nonionic surfactant that increases the solubility of hydrocarbons, thus increasing their bioavailability. Ferreira *et al*.^47^ registered the potential of *B. thuringiensis* strains isolated from marine sediments to degrade emerging pollutants such as PAHs and pesticides.

TPH degradation efficiency obtained by *Rhodococcus hoagii* was higher than values reported by previous authors and occurred in shorter incubation time. For example, Aboelwafa and Alwasify^48^ found that *Pseudomonas aeruginosa* degraded 53.68% of petroleum within five days, whereas *B. subtilis* reduced 47.35% and *Acinetobacter lwoffi* degraded 38.07% of petroleum during the same period. After 28 days, the degradation percentages were 77.8%, 76.7% and 74.3%, respectively, which are lower than the efficiency achieved with *R. hoagii* in 24 h (Supplementary Table S1).

The genus *Rhodococcus* presents ample capacity to transform and degrade various organic substrates, such as aliphatic and aromatic hydrocarbons, phenols, pesticides, coal and petroleum^49,50^. Thus, this genus has been considered promising for bioremediation of contaminated environments due to the high metabolic capacity and safety^49,50^. Species *Rhodococcus hoagii* was previously known as *Rhodococcus equi*, which has been reported as a fast and efficient in degradation of petroleum^51,52^. In the present study, *R. hoaggi*, despite presenting the lowest biomass content among the studied species, consumed over 85% of TPHs in 24 h. This result is higher than the efficiencies reported by Al-Surrayai *et al*.^52^, between 17.9% and 97% for different hydrocarbons after incubation of 2 weeks. Therefore, *R hoagii* metabolism can be considered an ideal strain for the biodegradation, using TPH as their sole carbon source.

## Conclusion

Three rhizospheric microorganisms, namely *Bacillus thurigiensis*, *Bacillus pumilus* and *Rhodococcus hoagii*, were successfully isolated from *Panicum aquaticum* Poir. cultivated in petroleum-contaminated soil. These species have been previously isolated from petroleum contaminated soils^41,46^ and applied in the degradation of petroleum or hydrocarbons^46,47,51,52^, however, the present work resulted in new strains, thus with different oil degradation efficiencies. The results reported here confirm the petroleum hydrocarbons degradation potential by these species, particularly *R. hoagii*, which consumed over 85% of TPH in 24 h, a higher and faster efficiency than those reported previously in the literature. Bioremediation is ruled by several parameters where the metabolism of selected microorganisms is essential to enable tolerance and degradation of the target pollutant. Future biotechnological products, such as bacterial consortia, can be developed to further improve the bioremediation of this problematic contaminant in soil and water.

## Supplementary information

Supplementary Table S1.

## References

[1] Van Hamme JD, Singh A, Ward OP (2003). Recent advances in petroleum microbiology. Microbiol. Mol. Biol. Rev..

[2] Abdel-Shafy HI, Mansour MSM (2016). A review on polycyclic aromatic hydrocarbons: Source, environmental impact, effect on human health and remediation. Egyptian J. Pet..

[3] Samanta SK, Singh OV, Jain RK (2002). Polycyclic aromatic hydrocarbons: environmental pollution and bioremediation. Trends in Biotech..

[4] Das K, Mukherjee AK (2007). Crude petroleum-oil biodegradation efficiency of Bacillus subtilis and Pseudomonas aeruginosa strains isolated from a petroleum-oil contaminated soil from North-East India. Bioresour. Technol..

[5] Gerhardt KE, Huang X, Glick BR, Greenberg B (2009). Phytoremediation and rhizoremediation of organic soil contaminants: potential and challenges. Plant Sci..

[6] Balachandran C, Duraipandiyan V, Balakrishna K, Ignacimuthu S (2012). Petroleum and polycyclic aromatic hydrocarbons (PAHs) degradation and naphthalene metabolism in Streptomyces sp. (ERI-CPDA-1) isolated from oil contaminated soil. Bioresour. Technol..

[7] Sloan, N. A. Oil impacts on cold-water marine resources: A review relevant to Parks Canada’s evolving Marine Mandate. *Occasional Paper* (2004).

[8] Wang J (2008). Phytoremediation of petroleum polluted soil. Pet. Sci..

[9] Pandey J, Chauhan A, Jain RK (2009). Integrative approaches for assessing the ecological sustainability of *in situ* bioremediation. FEMS Microbiol. Rev..

[10] Paul D, Pandey G, Pandey J, Jain RK (2005). Accessing microbial diversity for bioremediation and environmental restoration. Trends Biotechnol..

[11] Díaz E (2004). Bacterial degradation of aromatic pollutants: a paradigm of metabolic versatility. Int. Microbiol..

[12] Yanto D, Tachibana S (2014). Potential of fungal co-culturing for accelerated biodegradation of petroleum hydrocarbons in soil. J. Hazard. Mater..

[13] Leahy JG, Colwell RR (1990). Microbial degradation of hydrocarbons in the environment. Microbiol. Rev..

[14] Kuiper I, Lagendijk EL, Bloemberg GV, Lugtenberg BJJ (2004). Rhizoremediation: A beneficial plant-microbe interaction. Mol. Plant Microbe Interact..

[15] McCutcheon, S. C. & Schonnor, J. L. *Phytoremediation: transformation and control of contaminants*. John Wiley & Sons, New York (2003).

[16] Carneiro MAC, Siqueira JO, Moreira FMS (2001). Estabelecimento de plantas herbáceas em solo com contaminação de metais pesados e inoculação de fungos micorrízicos arbusculares. Pesq. Agropec. Bras..

[17] Merkl N, Schultze-Kraft R, Infante C (2005). Phytoremediation in the tropics e influence of heavy crude oil on root morphological characteristics of graminoids. Environ. Pollut..

[18] Ndimele PE (2010). A review on the phytoremediation of petroleum hydrocarbon. Pak. J. Biol. Sci..

[19] Chen Y-C, Banks MK, Schwab AP (2003). Pyrene degradation in the rhizosphere of tall fescue (Festuca arundinacea) and switchgrass (Panicum virgatum L). Environ. Sci. Technol..

[20] Huang LK, Bughrara SS, Zhang XQ, Bales-Arcelo CJ, Bin X (2011). Genetic diversity of switchgrass and its relative species in *Panicum* genus using molecular markers. Biochem. Syst. Ecol..

[21] Guglieri A, Longhi-Wagner HM, Zuloaga FO (2007). Panicum sect. Dichotomiflora (Hitchc. & Chase) Honda e P. sect. Virgata Hitchc. & Chase ex Pilg. (Poaceae: Panicoideae: Paniceae) no Brasil. Acta. Bot. Bras..

[22] Gamalero E, Lingua G, Berta G, Lemanceau P (2003). Methods for studying root colonization by introduced beneficial bacteria. Agronomie.

[23] Colle, J. G., Fraser, A. G., Marmion, B. P. & Simmons, A. *Practical Medical Microbiology*, 14th edn. Churchill Livingstone, New York (1996).

[24] Thomaz-Soccol V (2009). Casos alóctones de leishmaniose visceral canina no Paraná, Brasil: implicações epidemiológicas. Rev. Bras. Parasitol. Vet..

[25] Lane DJ, Field KG, Olsen GJ, Pace NR (1988). Reverse transcriptase sequencing of ribosomal RNA for phylogenetic analysis. Methods. Enzymol..

[26] Lane DJ (1985). Rapid determination of 16S ribosomal RNA sequences for phylogenetic analyses. Proc. Natl. Acad. Sci..

[27] Chaskes, S. & Austin, R. Stains for light microscopy, in: Goldman E, Green LH (eds) *Practical Handbook of Microbiology*. CRC Press, Boca Raton, pp. 45–60 (2015).

[28] Mishra S, Sarma PM, Lal B (2004). Crude oil degradation efficiency of a recombinant Acinetobacter baumannii strain and its survival in crude oil-contaminated soil microcosmo. FEMS Microbiol. Lett..

[29] Déziel E, Paquette G, Villemur G, Lépine F, Bisaillon JG (1996). Biosurfactant production by a soil Pseudomonas strain growing on polycyclic aromatic hydrocarbons. Appl. Environ. Microb..

[30] Omotayo AE, Ojo OY, Amund OO (2012). Crude oil degradation by microorganisms in soils composts. Res. J. Microbiol..

[31] Makkar RS, Cameotra SS (1998). Production of biosurfactant at mesophilic and thermophilic conditions by a strain of *Bacillus subtilis*. J. Ind. Microbiol. Biot..

[32] Schwab AP, Su J, Wetzel S, Pekarek S, Banks MK (1999). Extraction of petroleum hydrocarbons from soil by mechanical shaking. Environ. Sci. Technol..

[33] Callegari-Jacques S. M. *Bioestatística: Princípios e Aplicações*, 1st edition. Artmed, Porto Alegre (2003).

[34] Chen HJ, Guo GL, Tseng DH, Cheng CL, Huang SL (2006). Growth factors, kinetics and biodegradation mechanism associated with Pseudomonas nitroreducens TX1 grown on octylphenol polyethoxylates. J. Environ. Manage..

[35] Holliger C (2007). Contaminated environments in the subsurface and bioremediation: organic contaminants. FEMS Microbiol. Rev..

[36] Kanaly RA, Harayama S (2000). Biodegradation of high-molecular-weight polycyclic aromatic hydrocarbons by bacteria. J. Bacteriol..

[37] Deng M (2014). Isolation and characterization of a novel hydrocarbon-degrading bacterium Achromobacter sp. HZ01 from the crude oil-contaminated seawater at the Daya Bay, southern China. Mar. Pollut. Bull..

[38] Atekwana EA (2004). *In-situ* apparent conductivity measurements and microbial population distribution at a hydrocarbon-contaminated site. Geophysics.

[39] Zhang J (2014). Petroleum contamination of soil and water, and their effects on vegetables by statistically analyzing entire data set. Sci. Total Environ..

[40] Liu PG, Chang TS, Chen C, Wang M, Hsu H (2013). Effects of soil organic matter and bacterial community shift on bioremediation of diesel-contaminated soil. Int. Biodeter. Biodegr..

[41] Wolińska A (2018). Catabolic fingerprinting and diversity of bacteria in mollic gleysol contaminated with petroleum substances. Appl. Sci..

[42] Rahman PKSM, Rahman T, Lakshmanaperumalsamy P, Banat IM (2002). Occurrence of crude oil degrading bacteria in gasoline and diesel station soils. J. Basic Microb..

[43] Calvo C, Toledo FL, González-López J (2004). Surfactant activity of a naphthalene degrading Bacillus pumilus strain isolated from oil sludge. J. Biotechnol..

[44] Bento FM, Camargo FAO, Okeke BC, Frankenberger WT (2005). Diversity of biosurfactant producing microorganisms isolated from soils contaminated with diesel oil. Microbiol. Res..

[45] De J, Ramaiah N, Vardanyan L (2008). Detoxification of toxic heavy metals by marine bacteria highly resistant to mercury. Mar. Biotechnol..

[46] Maiti A, Das S, Bhattacharyya N (2013). Bioremediation of high molecular weight polycyclic aromatic hydrocarbons by Bacillus thuringiensis Strain NA2. *J*. Science.

[47] Ferreira L, Rosales E, Danko AS, Sanromán MA, Pazos MM (2015). Bacillus thuringiensis a promising bacterium for degrading emerging pollutants. Process. Saf. Environ..

[48] Aboelwafa AM, Alwasify RS (2009). Biodegradation of crude oil using local isolates. Aust J. Basic & Appl. Sci..

[49] Larkin MJ, Kulakov LA, Allen CCR (2005). Biodegradation and Rhodococcus - masters of catabolic versatility. Curr. Opin. Biotechnol..

[50] Kuyukina MS, Ivshina IB, Serebrennikova MK, Rubtsova EV, Krivoruchko AV (2013). Simultaneous species-specific PCR detection and viability testing of poly (vinyl alcohol) cryogel-entrapped *Rhodococcus* spp. after their exposure to petroleum hydrocarbons. J. Microbio. Meth..

[51] Ko SH, Lebeault JM (1999). Effect of a mixed culture on co‐oxidation during the degradation of saturated hydrocarbon mixture. J. Applied. Microbiol..

[52] Al-Surrayai T, Yateem A, Al-Kandari R, Al-Sharrah T, Bin-Haji A (2009). The use of Conocarpus lancifolius trees for the remediation of oil-contaminated soils. Soil Sediment. Contam..

