# Supplementary material for: Petroleum-Tolerant Rhizospheric Bacteria: Isolation, Characterization and Bioremediation Potential
Source: Sci Rep. 2020 Feb 6;10:2060. doi: 10.1038/s41598-020-59029-9 (PMC7005311; doi:10.1038/s41598-020-59029-9)
Supplement: Supplementary file 1 — Supplementary Table S1. [file 41598_2020_59029_MOESM1_ESM.pdf]

PETROLEUM-TOLERANT RHIZOSPHERIC BACTERIA: ISOLATION, CHARACTERIZATION AND BIOREMEDIATION POTENTIAL

Jéssica Aparecida Viesser[1], Maura Harumi Sugai-Guerios[1], Lucca Centa Malucelli[2], Marcia Regina Pincerati[1], Susan Grace Karp[1,3], Leila Teresinha Maranho[1*]

[1]PhD Program in Industrial Biotechnology, Universidade Positivo (UP), Av. Pedro Prof. Viriato Parigot de Souza, 5300, CEP 81280-330, Curitiba, PR, Brazil.

[2]PhD Program in Environmental Management, Universidade Positivo (UP), Av. Pedro Prof. Viriato Parigot de Souza, 5300, CEP 81280-330, Curitiba, PR, Brazil.

[3]PhD Program in Bioprocess Engineering and Biotechnology, Universidade Federal do Paraná, Caixa Postal 19011 – ACF Centro Politécnico, CEP 81531-980, Curitiba, PR, Brazil.

*Corresponding author: Leila Teresinha Maranho

E-mail: maranho@up.edu.br

Supplementary Table S1. Peak areas and corresponding degradation efficiency of petroleum hydrocarbons by *Bacillus thuringiensis, Bacillus pumilus* and *Rhodococcus hoagii* during cultivation in mineral medium with 1% (v/v) of petroleum.

	**CULTIVATION TIME (h)**	**RETENTION TIME (min)**	**6.12**	**7.28**	**8.22**	**9.02**	**9.49**	**9.75**	**10.19**	**10.44**	**11.98**	**12.68**	**13.49**	**17.29**
Bacillus thuringiensis	**24**	AREA (C)	15782.77	38906.33	60380.02	83207.12	36547.58	58675.58	26444.62	48879.57	34548.21	22417.89	20989.01	24883.68
		AREA (B)	8424.58	24444.54	44438.62	57604.08	27004.13	41973.87	14474.14	35019.46	25410.60	17263.02	16343.81	19827.73
		**DEGRADATION (%)**	**46.62**	**37.17**	**26.40**	**30.77**	**26.11**	**28.46**	**45.27**	**28.36**	**26.45**	**22.99**	**22.13**	**20.32**
	**48**	AREA (C)	4905.07	25917.59	63515.81	95475.17	43131.57	71962.57	39507.89	60958.71	51005.34	32492.99	34423.26	43916.42
		AREA (B)	452.67	5972.43	19472.48	38091.19	19766.17	31593.69	13208.23	27516.14	19681.03	13188.38	11988.24	12304.80
		**DEGRADATION (%)**	**90.77**	**76.96**	**69.34**	**60.10**	**54.17**	**56.10**	**66.57**	**54.86**	**61.41**	**59.41**	**65.17**	**71.98**
Bacillus pumilus	**24**	AREA (C)	10663.17	23245.13	65767.27	101276.20	48018.77	75813.47	41486.70	66172,53	49020.43	32751.57	31933.67	47212.90
		AREA (B)	2857.60	16857.42	52795.18	63709.86	28286.08	43704.79	33271.81	43154.32	26825.43	24572.39	17106.96	27245.59
		**DEGRADATION (%)**	**73.20**	**27.48**	**19.72**	**37.09**	**41.09**	**42.35**	**19.80**	**34.79**	**45.28**	**24.97**	**46.43**	**42.29**
	**48**	AREA (C)	18209.40	60414.67	115078.77	150947.84	66199.36	105736.79	32022.02	89253.21	62113.70	42306.98	41941.01	54706.94
		AREA (B)	4450.33	21106.70	46895.30	65256.58	27790.12	46427.70	24640.80	34917.81	23650.00	16489.26	14795.12	18342.97
		**DEGRADATION (%)**	**75.56**	**65.06**	**59.25**	**56.77**	**58.02**	**56.09**	**23.05**	**60.88**	**61.92**	**61.02**	**64.72**	**66.47**
Rhodococcus hoagii	**24**	AREA (C)	33812.67	143947.44	317789.33	449380.41	209827.22	334562.72	195664.16	282753.68	242656.64	188496.18	224233.72	362542.30
		AREA (B)	2799.77	14717.26	37863.94	59186.74	29767.46	46250.74	25289.14	35413.91	28986.10	21615.08	22678.41	52234.62
		**DEGRADATION (%)**	**91.72**	**89.78**	**88.09**	**86.83**	**85.81**	**86.18**	**87.08**	**87.48**	**88.05**	**88.53**	**89.89**	**85.59**
	**48**	AREA (C)	18892.59	96278.82	249267.26	405706.81	197516.02	321073.89	193629.83	276925.86	250323.31	199285.22	235855.00	375534.21
		AREA (B)	727.92	7192.09	23691.18	42964.57	26048.33	35239.68	23218.17	27082.53	27423.67	17639.81	18562.31	53197.27
		**DEGRADATION (%)**	**96.15**	**92.53**	**90.50**	**89.41**	**86.81**	**89.02**	**88.01**	**90.22**	**89.04**	**91.15**	**92.13**	**85.83**

* (C): Treatment Control; (B): Treatment with bacteria.
